# Topical immunotherapy of severe alopecia areata with diphenylcyclopropenone (DPCP): experience in an Iranian population

**DOI:** 10.1186/1471-5945-5-6

**Published:** 2005-05-26

**Authors:** Shahin Aghaei

**Affiliations:** 1 Department of Dermatology, Jahrom Medical School, Jahrom, Iran

## Abstract

**Background:**

Highly variable results of topical diphenylcyclopropenone (DPCP) in the treatment of alopecia areata have been reported so far. The purposes of the present study were to evaluate the efficacy and tolerability of DPCP treatment in severe alopecia areata.

**Methods:**

Twenty-eight patients (16 female and 12 male, 10–35 years old, mean age 25 years) with extensive alopecia areata were enrolled in an open-label clinical trial. After sensitization with 2% DPCP, progressively higher concentrations beginning at 0.001% were applied weekly for 6 months to one side of the scalp, after which, if terminal hair growth was noted, the entire scalp was then treated under the same weekly protocol. The maximum concentration of DPCP in acetone was 2%.

**Results:**

Twenty-seven of 28 patients completed therapy. The overall response rate was 81.5% (22/27), complete remission (90%-100% terminal hair re-growth) was obtained 22.2% (6/27) and partial remission (10%-90% terminal hair re-growth) in 59.3% (16/27). In all patients an eczematous reaction consisting of erythema, itching, and scaling at the site of application were observed. During therapy, other side effects including, occipital lymphadenopathy 40.7% (11/27), severe eczema/blister formation 40.7% (11/27), hyperpigmentation 18.5% (5/27) were observed, but no hypopigmentation, vitiligo, contact urticaria, and erythema multiforme-like reaction were seen in the patients. Nineteen of 27 (70.4%) patients had at least one side effect, other than eczematous reaction. Notably, partial recurrence was observed in 50.9% (13/22) of these patients after 6 to 12 months of follow-up. During the follow-up time the maintenance DPCP immunotherapy was continued.

**Conclusion:**

Topical DPCP treatment for alopecia areata is an effective therapy with a slightly high relapse rate during bilateral maintenance treatment. According to the author's knowledge this is the first experience with DPCP in Iran.

## Background

Dermatosis of the scalp such as alopecia areata (AA) are a burden for many patients and often resistant, even to extensive therapy [1-4]. Furthermore, treatment results can be difficult to interpret because of spontaneous remissions and recurrences, as well as the use of several therapies simultaneously, such as tretinoin creams, minoxidil lotions and zinc supplementation [4]. Topical and intra-lesional corticosteroid therapy is frequently tried, but the benefit of such treatment is often questionable or temporary. Systemic corticosteroid treatment may be effective in some cases, but the maintenance dose needed is often high [4,5]. Some success, has been reported with anthralin, but results seem variable [6,7]. Other therapies which have been tried, with variable success, include minoxidil [8,9], cyclosporine [10-12], alpha-interferon [13], and acupuncture [14].

Topical immunotherapy has been used for more than 10 years for the treatment of severe AA and various contact allergens such as dinitrochlorobenzene (DNCB), squaric acid dibutylester (SADBE), and diphenylcyclopropenone (DPCP) have determined re-growth of hair in patients with AA [15-17].

The effectiveness of topical immunotherapy with AA has been demonstrated in several reports, although the response rate varied greatly from 4% to 85% [18]. The present study was performed as an open-label clinical trial to evaluate the efficacy and tolerability of topically administered DPCP in patients with extensive AA. In addition, the clinical response with prognostic factors was correlated and assessed the patients' response after a follow-up of 6 to 12 months, in which bilateral DPCP maintenance treatment was continued.

## Methods

During 2 years between April 2001 and May 2003 at the Department of Dermatology, Saadi Hospital, Shiraz University of Medical Sciences, 28 patients (12 men, 16 women) with severe AA (> 40% scalp hair loss) were enrolled in an open-label clinical trial. Their ages ranged from 10 to 35 years (mean, 25 years). The DPCP used (Acros Organics, New Jersey, USA) 98% pure, was dissolved in acetone. Informed consent was obtained. Women of childbearing age were required to use a reliable form of birth control. Individuals with AA were ineligible for DPCP treatment if they presented with less than 40% scalp involvement, the age less than 10 years, significant cardiovascular disease, pregnancy, or serious intercurrent medical illnesses. In brief, sensitization was performed with a 2% solution of DPCP applied to an area of 5× 5 cm on one side of the scalp. Two weeks following sensitization, treatment was started by weekly ipsilateral applications of incremental concentrations of DPCP (between 0.001% and 2%) adjusted to the patient's reactivity to the contact allergen. The aim was to maintain mild contact eczema and itch for about 48 hours after application. Patients were instructed to avoid direct sun exposure of the scalp and not to wash the scalp for 48 hours after each application. The opposite half of the scalp served as a control to rule out possible spontaneous remission. Once hair re-growth occurred on the treated side, the applications were extended over the entire scalp. The overall duration of therapy varied from 6 to 12 months. The patients, who achieved clinically significant re-growth, were under follow-up of 6–12 months, with ongoing maintenance DPCP immunotherapy.

The types of AA before treatment were classified as follows: (1) multilocular AA including ophiatic type with > 40% loss of scalp hair; (2) subtotal or total AA; (3) AA partim universalis (loss of all scalp hair with some body hair left) or universalis (loss of all scalp and body hair). According to the response to topical DPCP immunotherapy, patients were grouped into 3 categories: no hair re-growth (< 10% terminal hair), partial hair re-growth (10–90% terminal hair) and complete hair re-growth (90–100% terminal hair).

Efficacy evaluation was performed with clinical examination. If no re-growth was observed within 6 months of treatment, the patient was considered to be a non-responder and was dropped from the trial.

In all patients, the following laboratory tests were performed at baseline, every 3 months during treatment and after treatment: complete blood cell count; chemistry profile; urinalysis; levels of thyroid hormones (free tri-iodothyronine, free thyroxine, thyroid stimulating hormone); fasting blood sugar; and antinuclear antibody titres (ANA). Other laboratory tests such as anti-gastric parietal cells, anti-thyroglobulin and anti-smooth muscle antibody titres were not done, because of loss of these capabilities in our institute.

The influence of the following factors on the outcome of topical immunotherapy were investigated: sex, age at onset, atopy, disease duration before treatment, type of AA, presence of nail changes, presence of naevus simplex, and family history of AA and other auto-immune disorders such as diabetes mellitus (DM) and thyroid diseases. To assess any correlations between the above mentioned parameters the X^2 ^test was used.

## Results

Twenty-seven out of 28 patients completed the treatment. The demographic and clinical data of the patients included in the study are summarized in table 1 and 2.

**Table 1:** Demographic data

*Number of patients*	27
*Sex M/F*	12/15
*Age (range, mean)*	10–35, 25 years
*Disease duration before treatment (range, mean)*	0.58–28, 5.82 years

**Table 2:** Clinical data

***Type of AA***	
(1) Multi-locular including ophiatic type (>40% of scalp hair loss)	11/27 (40.7%)
(2) Subtotal, total	5/27 (18.6%)
(3) Universalis, partim universalis	11/27 (40.7%)

***Positive family history of:***	

AA	10/27 (37%)
Thyroid disease	7/27 (6%)
DM	9/27 (33.3%)

***Positive personal history of:***	

Thyroid disease	3/27 (11.1%)
DM	None
***Presence of nail changes***	16/27 (59.3%)
***Presence of naevus simplex on the posterior neck***	11/27 (40.7%)
***Circulating ANA***	None

Disease duration before treatment ranged from 0.58 (7 months) to 28 years. The duration of therapy ranged from 6 to 24 months, including long-term patients with repeated DPCP treatment for maintaining hair re-growth.

Overall 81.5% (22 of 27 patients) responded to therapy: 22.2% (6 of 27 patients) achieved complete hair re-growth (90–100% terminal hair), and 59.3% (16 of 27 patients) had partial hair re-growth (10–90% terminal hair). Five patients had no hair re-growth (18.5%). Of the 22 patients with complete and partial remission, 13 (50.9%) suffered a relapse either simultaneously maintenance treatment of follow-up or following termination of therapy. No abnormalities were detected on baseline and follow-up laboratory data in these patients.

Side effects following sensitization were seen in 19 of 27 patients (70.4%): occipital lymphadenopathy in 40.7% (11 of 27 patients), severe eczema/blister formation in 22.2% (6 of 27 patients), and hyperpigmentation in 18.5% (5 of 27 patients). No cases of hypopigmentation, vitiligo, contact urticaria, and erythema multiforme-like reaction were observed. The therapeutically induced mild contact eczema with itching was not considered as adverse effects.

## Discussion

Topical immunotherapy, using DPCP, is currently considered the most effective mode of treatment. However, the way in which DPCP operates on hair follicles in AA still remains to be elucidated. Vascular endothelial growth factor (VEGF), essential for angiogenesis and vascular permeability, may be responsible for maintaining proper vasculature around hair follicles, and several studies provide evidence that apoptosis is a central element in the regulation of hair follicle and vascular regression [19]. Moreover, topical immunotherapy considerably alters the peribulbar CD4/CD8 ratio in human and experimental animal studies, restoring a condition close to normal scalp skin [19,20].

In the present study, growth of terminal hair on the entire scalp was achieved totally in 81.5% of patients with AA after 6–12 months of treatment with DPCP. This results are less than those reported by Cotellessa et al [18] who observed a 48% complete success rate in a series of 52 patients, and those of Weise et al [21] and Van der Steen et al [22] who detected 40% and 50.4% complete re-growth in 124 and 139 patients, respectively (complete hair re-growth in the present study was 22.2%). In other reports, however, the percentage of success greatly varied from 4% to 85% [17,21,23-25]. The discrepancy of response rates may be due to the number of patients in clinical trials; the type, duration, and severity of the AA; and different methods of assessing clinical efficacy.

In the present study, concomitant spontaneous re-growth of eyebrows and body could be observed in approximately half of the successfully treated patients, which are similar to those reported by Cotellessa et al [18]. Nine of 27 patients had positive family history of atopy, but no significant correlations between its frequency and treatment response revealed (P = 0.07). Also, positive personal/family history of thyroid disease, atopy, and diabetes mellitus (DM); sex; age of patients; type of AA; and duration of disease before treatment had no significant correlations with treatment response (P > 0.05). In addition, the X^2 ^test revealed significant correlation between the presence of naevus simplex (salmon patch) on the posterior neck and less favorable treatment response (P = 0.03). The same correlation between the presence of nail changes (such as pitting, ridging, leukonychia punctata, brittleness, "red spotted lunula", "sandpaper nails", and onychomadesis) and less favorable response rate was detected (P = 0.02). Remarkably, with regard to patients' follow-up with ongoing maintenance therapy in patients with significant hair re-growth, partial recurrences of AA were observed in about one-half of the patients.

Wiesman et al. referred to the risks of relapse at long-term follow-up. After 35 months of follow-up, 62.6% of the subjects who had been successfully treated experienced a relapse, and this risk was not influenced by the implementation of maintenance therapy [26]. This result is in agreement with the present study. But, other investigators believe that if this result could be reproduced in other studies, the patient could stop treatment when hair re-growth was complete because maintenance treatment would be of no use [27].

Among the investigated "prognostic factors" for the outcome of DPCP treatment, which have formerly been studied with variable results [21,25,28], only the presence of naevus simplex and nail changes were found to be of significance.

The main side effects observed during therapy with relatively high frequency were severe eczema/bullous formation, occipital lymphadenopathy, and hyperpigmentation. In none of the patients hypo-pigmentation or vitiligo were observed.

## Conclusion

The study findings are in agreement with former studies showing the efficacy of topical immunotherapy with DPCP in the treatment of severe AA, but with a slightly high relapse rate during bilateral maintenance treatment. According to the author's knowledge this is the first experience with DPCP in Iran.

## List of abbreviations used

AA: Alopecia areata

DPCP: Diphenylcyclopropenone

DNCB: Dinitrochlorobenzene

SADBE: Squaric acid dibutylester

DM: Diabetes mellitus

ANA: Antinuclear antibody test

VEGF: Vascular endothelial growth factor

## Competing interests

The author declares that he has no competing interests.

**Figure 1:**
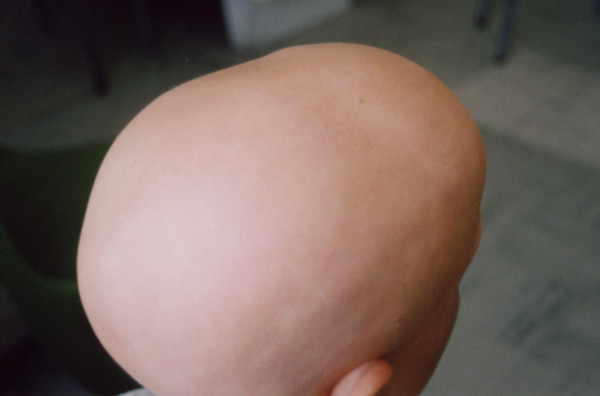
A 12-year-old patient with alopecia totalis before treatment.

**Figure 2:**
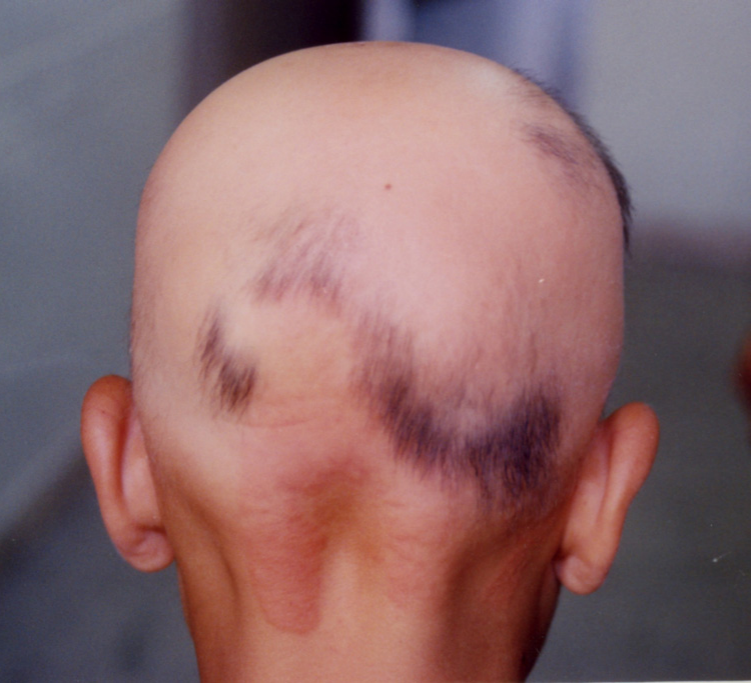
The same patient 4-weeks after treatment.

**Figure 3:**
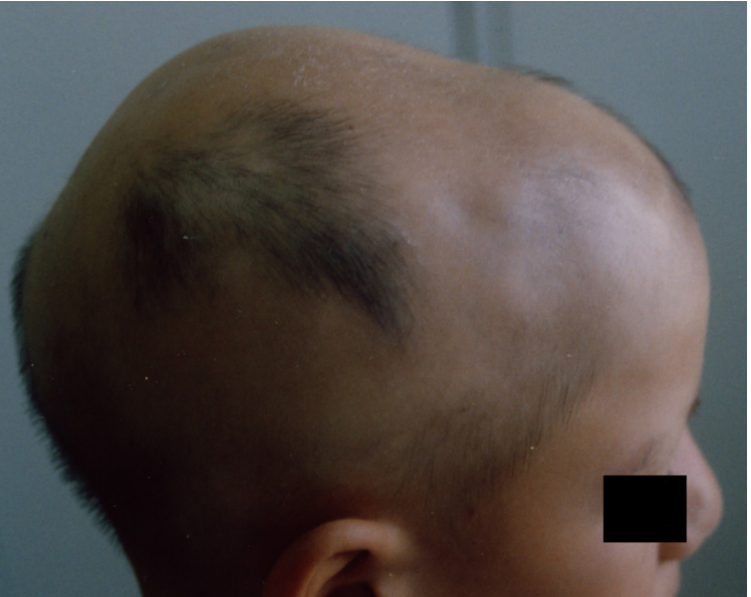
After 20-weeks of treatment (bilateral treatment started on 12-weeks, no photo available).

**Figure 4:**
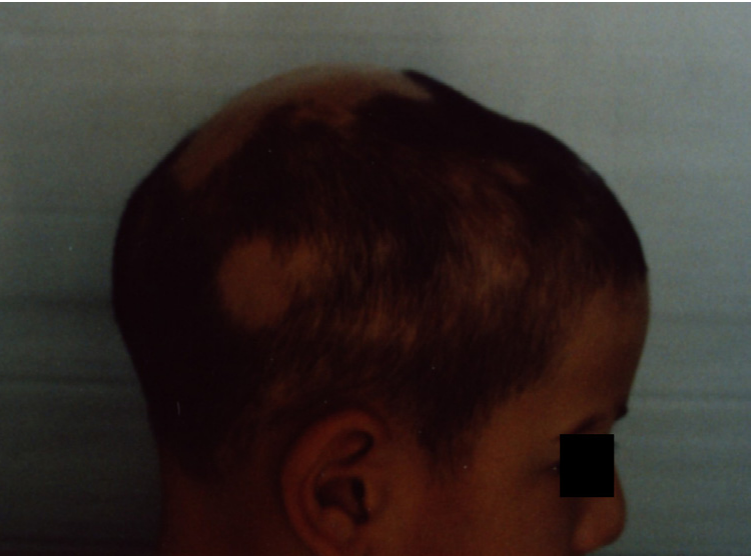
After 24-weeks of treatment (right view).

**Figure 5:**
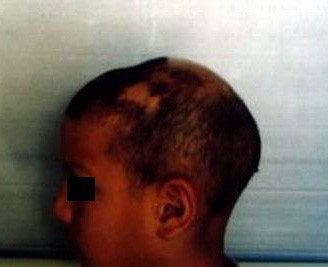
After 24-weeks of treatment (left view).

**Figure 6:**
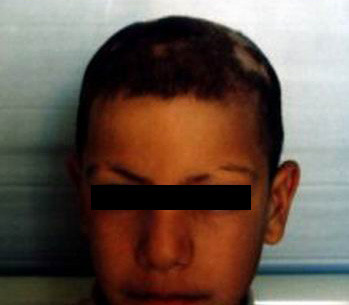
After 28-weeks of treatment (front view).

## Pre-publication history

The pre-publication history for this paper can be accessed here:


